# A Rare Case of Lipoid Proteinosis in a Patient Presenting With Seizures: A Case Report and Literature Review

**DOI:** 10.7759/cureus.73904

**Published:** 2024-11-18

**Authors:** Chandrashekar Patil, Bhagwat Kadam, Sachin D Dawale, Saiprasad P Shelke, L Ramitha

**Affiliations:** 1 Radiology, Malla Reddy Medical College for Women, Hyderabad, IND; 2 Radiodiagnosis, Malla Reddy Medical College for Women, Hyderabad, IND; 3 Radiology, Mahatma Gandhi Institute of Medical Sciences, Sevagram, IND; 4 Radiology, Seth Gordhandas Sunderdas Medical College and King Edward Memorial Hospital, Mumbai, IND; 5 Paediatrics, S Nijalingappa Medical College and Hanagal Shree Kumareshwar (HSK) Hospital and Research Centre, Bagalkot, IND

**Keywords:** amygdala, calcification, extracellular matrix protein, lipoid proteinosis, seizures, temporal lobe, urbach-wiethe disease

## Abstract

Lipoid proteinosis is a rare genetic disorder affecting the skin, mucous membranes, and central nervous system. Here, we present the case of a 35-year-old female who presented with two episodes of seizures followed by loss of consciousness and injury to the nose. A CT scan and MRI of the brain revealed small symmetrical calcifications in the bilateral medial temporal lobes, a finding highly suggestive of lipoid proteinosis. This case highlights the neurological manifestations of lipoid proteinosis, an extremely rare autosomal recessive disorder, and emphasizes the importance of neuroimaging in its diagnosis.

## Introduction

Lipoid proteinosis (LP), also known as Urbach-Wiethe disease, is a rare autosomal recessive genetic disorder characterized by the deposition of hyaline material in various tissues, including the skin, mucous membranes, and central nervous system. LP is caused by mutations in the extracellular matrix protein 1 (*ECM1*) gene [1]. Although the exact function of the *ECM1* gene is not known, it is thought to have important physiological and biological actions in epidermal differentiation, binding of dermal collagens and proteoglycans, and regulation of angiogenesis. Cutaneous lesions such as blisters usually develop within the first few years of life, though the timing of their onset is variable [2]. The disease is slowly progressive and tends to have a benign course [3]. Brain involvement, particularly symmetrical calcifications in medial temporal lobes, is the hallmark of the disease and can lead to seizures and other neurological symptoms. This case report illustrates a patient who presented with seizures as the initial manifestation of LP, underscoring the need for awareness of this condition among clinicians.

## Case presentation

The patient presented to the emergency department with complaints of two seizure episodes occurring two hours apart. Each seizure episode was characterized by a sudden onset, with the patient losing consciousness for approximately 10 minutes after the second seizure. During the second seizure, the patient sustained a nasal injury, resulting in pain, with swelling noted over the nose. There was no history of frothing, involuntary urination, or defecation during the seizures. There was no associated fever or recent history of illness. On examination, the patient was alert but anxious. The swelling and tenderness over the bridge of the nose were consistent with recent trauma.

No focal neurological deficits were noted. Cranial nerve examination was unremarkable, and motor and sensory functions were intact. No obvious skin lesions or thickening were noted during the initial examination. Vital signs were within normal limits.

**Figure 1:**
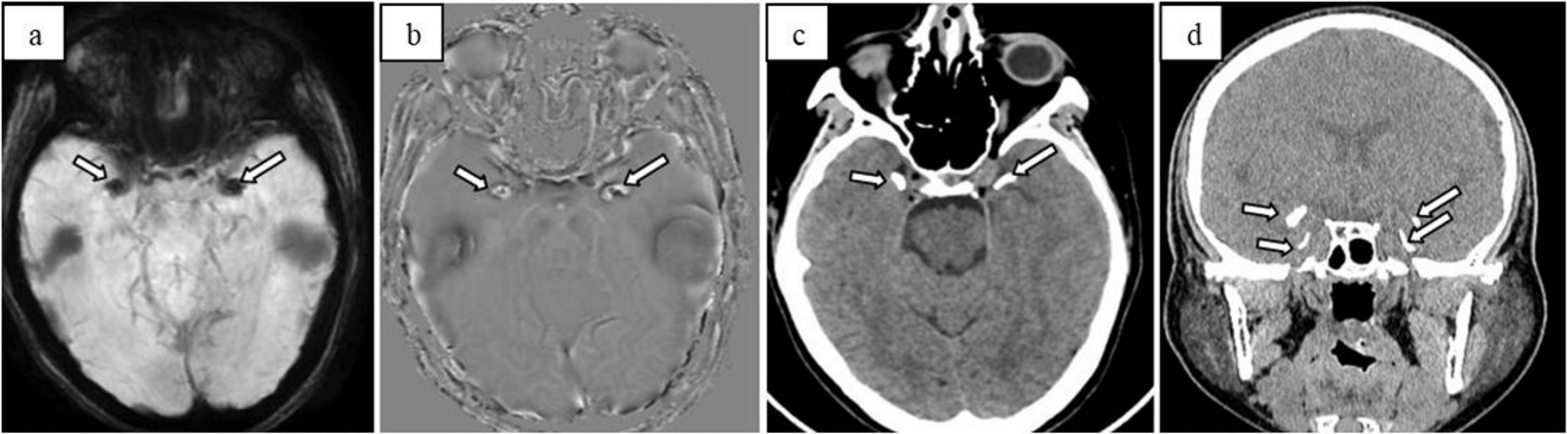
(a, b) susceptibility image showing blooming in the bilateral symmetrical medial temporal lobes with a corresponding bright signal on phase image (arrows), suggestive of calcifications. (c) Axial and (d) coronal CT images showing bilateral symmetrical medial temporal lobe calcifications (arrows). Note that the calcifications are bean and horn-shaped.

The patient’s investigation results, including a complete blood count, serum electrolytes, renal and liver function tests, and an electrocardiogram, were all within normal limits. However, the CT and MRI scan of the brain revealed small bean-shaped symmetrical calcifications in the bilateral medial temporal lobes (Figures 1a-1d), highly suggestive of LP, particularly given the patient’s seizure activity. Consequently, a provisional diagnosis of LP was made, with the temporal lobe calcifications likely contributing to the seizures.

The differential diagnosis included neurocysticercosis, tuberous sclerosis complex, idiopathic epilepsy, Fahr’s disease, and LP. For management, the patient was started on levetiracetam 500 mg twice daily to control seizure activity. Conservative treatment was provided for a nasal injury, including analgesics for pain relief and cold compresses for swelling. Neurological follow-up was arranged with a referral to a neurologist for further evaluation and long-term management of the seizure activity. Additionally, a referral to a dermatologist was made for a thorough examination to identify any subtle skin or mucosal involvement that could further support the diagnosis of LP. Although genetic testing for *ECM1* mutations was considered, it was deferred due to financial constraints. The patient was advised on the genetic nature of the disease and counseled about the possibility of future family screening.

At a three-month follow-up visit, the patient reported no further seizure activity since starting antiepileptic medication. The nasal injury had healed without complications. The patient continued to be monitored regularly by both neurology and dermatology specialists.

## Discussion

Vienna dermatologist and otorhinolaryngologist Urbach and Wiethe first described LP in 1929 by originally using the term “lipoidosis cutis et mucosae.” Neurological symptoms, especially seizures, are frequently linked to calcifications in the bilateral medial temporal lobes, as observed in this patient. Although dermatological signs are more prevalent, neurological manifestations can occasionally present as the first indication of the disease, as demonstrated in this case. Loss-of-function mutations in the *ECM1* gene, located on 1q21.2, lead to LP. Although the detailed function of the *ECM1* gene is not completely known, it is thought to play important roles in epidermal differentiation, binding with proteoglycans and dermal collagens, and regulating angiogenesis [1]. Glycoprotein *ECM1* has three types, namely, *ECM1a*, *ECM1b*, and *ECM1c*. *EMC1* is expressed in the dermis, basal keratinocytes, endothelial cells, and developing bones, linked to keratinocyte differentiation, basement membrane regulation, collagen composition, and growth factor binding [2]. LP patients are known to have calcifications in the bilateral temporal lobes or hippocampi in the brain [3]. Neurological manifestations of the disease, such as epilepsy and neuropsychiatric disorders, can be related to the inhibition by *ECM1* of MMP-9 activity, a 10a protein that is strongly expressed in the brain [4]. The calcifications in the temporal lobes are symmetrical and bilateral, presenting as horn-shaped and bean-shaped structures that affect the amygdala nuclei in the uncus. This is attributed to infiltration of the hippocampus along with perivascular calcium deposits and gliosis. Patients with a longer disease duration show more pronounced involvement of the bilateral amygdalae [4]. The involvement of the amygdala nuclei in the uncus is pathognomonic for this disease [5]. This is usually more evident in patients with longstanding LP [6]. The calcifications tend to involve the uncinate and parahippocampal gyri [7]. Seizure activity in these patients may result from the calcifications disrupting normal neuronal function. LP patients tend to have an increased incidence of mood, anxiety, and psychotic disorders. The patients also have varying degrees of mental retardation, as well as disturbances in decision-making and memory and abnormal social interaction [8]. Generalized dystonia has also been reported in LP. Dysfunction of the amygdala in many affected individuals may result in neuropsychiatric pathology [9]. Generally, neuropathological analysis following death shows calcium deposits, adjacent gliosis, vessel calcification, occlusions leading to perivascular infarctions, and evidence of demyelination. Bayakal et al. concluded in their case series of 14 patients that bilateral mesial temporal lobe calcification is diagnostic of LP [10].

Management of LP is largely symptomatic, focusing mainly on controlling seizures and addressing other clinical manifestations. However, LP typically follows a benign, slowly progressive course with a normal lifespan. Antiepileptic drugs, such as levetiracetam, are effective in controlling seizures, although the overall prognosis varies depending on the extent of neurological involvement.

## Conclusions

This case report underscores the importance of considering LP in patients presenting with seizures and characteristic bilateral symmetric medial temporal lobe calcifications on CT or MRI. While skin and mucosal changes are classic indicators of LP, neurological symptoms can sometimes be the first clinical signs, underscoring the importance of considering LP in patients with unexplained medial temporal lobe calcifications and seizure activity. Hence, early radiological diagnosis is very important, as it enables timely intervention to prevent further neuropsychiatric complications and allows for genetic counseling about the hereditary nature of the disease. Further research into the pathophysiology of LP and its neurological manifestations may provide insights into more targeted therapeutic approaches and ultimately improve patient outcomes.
